# Auditory dysfunction and perinatal risk factors in high-risk infants: Insights from brainstem evoked response audiometry

**DOI:** 10.6026/973206300213715

**Published:** 2025-10-31

**Authors:** Mahima Sharma, Madhuri Sharma, Raghav Mehta, Arpana Singhal, Deepandra Garg

**Affiliations:** 1 Department of Physiology, RUHS College of Medical Sciences, Jaipur, Rajasthan, India; 2 Department of ENT, RUHS College of Medical Sciences, Jaipur, Rajasthan, India; 3 Department of Obstetrics & Gynaecology, RUHS College of Medical Sciences, Jaipur, Rajasthan, India; 4 Department of Paediatrics, RUHS College of Medical Sciences, Jaipur, Rajasthan, India

**Keywords:** Hearing loss, brainstem evoked response audiometry, high-risk infants, perinatal risk factors, newborn screening, auditory neuropathy

## Abstract

Congenital hearing impairment is a major developmental disability, especially among infants with perinatal risk factors. This cross-sectional study evaluated 150 high-risk neonates in a NICU using Brainstem Evoked Response Audiometry (BERA). Abnormal BERA findings were observed in 30% of infants, with significant associations to severe hyperbilirubinemia, perinatal asphyxia and prolonged mechanical ventilation (p<0.001). Mean wave V latency was notably prolonged in infants with abnormal results. Thus, we show the importance of universal newborn hearing screening, particularly in high-risk groups, to enable early diagnosis and timely intervention.

## Background:

Hearing is a fundamental sense for the development of speech, language and cognitive skills in early childhood [1]. The absence of adequate auditory stimulation during the critical first few years of life can lead to irreversible deficits in communication abilities and social integration. The global incidence of significant bilateral congenital hearing loss is estimated to be 1 to 3 per 1000 live births in the general population; however, this figure rises dramatically to 2 to 4 per 100 in infants admitted to a Neonatal Intensive Care Unit (NICU) [2]. This increased vulnerability is attributed to their exposure to a multitude of perinatal and postnatal risk factors that can damage the delicate structures of the auditory system. The Joint Committee on Infant Hearing (JCIH) has established a list of risk indicators for congenital and early-onset hearing loss, which includes factors such as a family history of hearing loss, in-utero infections, craniofacial anomalies, low birth weight, prematurity, hyperbilirubinemia, perinatal asphyxia, exposure to ototoxic medications and prolonged mechanical ventilation [3]. These factors can affect the auditory system at various levels, from the outer ear to the auditory cortex, causing conductive, sensorineural, or neural hearing loss, including Auditory Neuropathy Spectrum Disorder (ANSD) [4]. Objective assessment of hearing in neonates is essential, as behavioral methods are unreliable. While Otoacoustic Emissions (OAEs) are widely used for universal screening and assessing cochlear outer hair cell function, they may miss neural deficits like ANSD. Brainstem Evoked Response Audiometry (BERA), also known as Auditory Brainstem Response (ABR), is the gold-standard electrophysiological test for this population [5]. It provides a non-invasive, objective measure of the entire auditory pathway from the cochlear nerve to the inferior colliculus in the brainstem, making it invaluable for both threshold estimation and neurodiagnostic assessment [6]. BERA records a series of stereotyped waves (I-V) that represent the sequential activation of neural generators along the auditory brainstem pathway. Abnormalities in the presence, latency, or amplitude of these waves can indicate the site and nature of the auditory lesion [7]. Numerous studies have investigated the link between perinatal risk factors and hearing loss. For example, severe hyperbilirubinemia has been strongly implicated due to the neurotoxic effects of unconjugated bilirubin on the auditory brainstem nuclei [8]. Similarly, perinatal asphyxia can lead to hypoxic-ischemic damage to the cochlea and central auditory pathways [9]. However, the prevalence of hearing loss and the relative importance of specific risk factors can vary significantly across different geographic regions and healthcare settings, influenced by local obstetric and neonatal care practices [10]. While the association between a high-risk status and hearing impairment is well-established, there remains a need for continuous evaluation of local risk profiles to refine screening protocols and allocate resources effectively. Understanding the specific constellation of factors that pose the greatest threat in a given population can help clinicians prioritize infants for diagnostic follow-up and counseling. Therefore, this study was designed to conduct a comprehensive assessment of auditory function using BERA in a cohort of high-risk infants admitted to a tertiary NICU. Therefore, it is of interest to determine the prevalence of abnormal BERA findings and to identify the key perinatal risk factors significantly associated with auditory dysfunction in this population.

## Materials and Methods:

## Study design and setting:

This was a cross-sectional analytical study conducted in the Department of Otorhinolaryngology in collaboration with the Neonatal Intensive Care Unit (NICU) of a tertiary care university hospital. The study was conducted over a period of 18 months, from January 2022 to June 2023.

## Study population and sample size:

The study population comprised infants admitted to the NICU who met at least one of the high-risk criteria for hearing loss as defined by the 2019 JCIH position statement. A total of 150 consecutive infants who fulfilled the selection criteria were enrolled. The sample size was determined based on an expected prevalence of hearing loss of 25% in the high-risk population, with a 95% confidence level and an 8% margin of error.

## Inclusion criteria:

Infants were included if they had one or more of the following risk factors:

[1] Gestational age <37 weeks (prematurity).

[2] Birth weight <2500 grams (low birth weight).

[3] NICU stay of >5 days.

[4] Hyperbilirubinemia requiring exchange transfusion or exceeding the threshold for phototherapy based on gestational age.

[5] Perinatal asphyxia (defined as an APGAR score of ≤5 at 5 minutes).

[6] Exposure to ototoxic medications (*e.g.*, aminoglycosides for >5 days, loop diuretics).

[7] Mechanical ventilation for >5 days.

[8] Family history of permanent childhood hearing loss.

## Exclusion criteria:

Infants were excluded from the study if they had major craniofacial anomalies affecting the external or middle ear, a confirmed diagnosis of a syndrome known to be associated with hearing loss (*e.g.*, Down syndrome, Waardenburg syndrome), active sepsis or clinical instability at the time of testing, or if parental consent was not provided.

## Data collection:

Detailed demographic and clinical data for each infant were collected from maternal and neonatal medical records using a pre-designed proforma. The variables recorded included maternal history, gestational age, birth weight, gender, APGAR scores at 1 and 5 minutes, duration of NICU stay, details of hyperbilirubinemia and its management, duration of mechanical ventilation and exposure to ototoxic drugs.

## Brainstem evoked response audiometry (BERA) procedure:

BERA testing was performed on all infants once they were medically stable and before hospital discharge. The procedure was conducted in a quiet, electrically shielded room with the infant in a state of natural sleep or after feeding. No sedation was used. An intelligent evoked potential system (Nihon Kohden Neuropack MEB-9200) was used for the BERA recording. The infant's skin was cleaned with a mild abrasive gel to ensure low electrode impedance (<5 kΩ). Silver-silver chloride disc electrodes were placed in a standard montage: the active electrode on the high forehead at the vertex (Cz), the reference electrode on the ipsilateral mastoid (Ai) and the ground electrode on the contralateral mastoid (Ac). Acoustic stimuli were delivered via insert earphones (ER-3A). The stimulus consisted of a 100-µs click presented monaurally. The test began with a screening intensity of 35 dB normal hearing level (nHL). If no response was obtained, the intensity was increased in 10-20 dB steps up to a maximum of 85 dB nHL to determine the hearing threshold. The stimulus repetition rate was 21.1 clicks per second. The responses were filtered (100-3000 Hz band-pass) and a total of 1500-2000 sweeps were averaged for each ear to obtain a clear and replicable waveform. Each test was repeated to ensure the reliability of the waveforms.

## Outcome measures:

The primary outcome was the BERA result, classified as either 'Normal' or 'Abnormal'.

[1] Normal BERA: The presence of replicable waves I, III and V at 85 dB nHL with absolute and interpeak latencies within the normal range for the infant's post-conceptional age. A click threshold of ≤35 dB nHL was considered a pass.

[2] Abnormal BERA: Characterized by one or more of the following: (i) an elevated threshold >35 dB nHL, indicating hearing loss; (ii) absence of any or all waves at the maximum intensity (85 dB nHL); (iii) significantly prolonged absolute or interpeak latencies beyond 2 standard deviations of the age-corrected norm.

## Statistical analysis:

Data were entered into Microsoft Excel and analyzed using SPSS Statistics for Windows, Version 25.0 (IBM Corp., Armonk, NY). Descriptive statistics were used to summarize the data, with continuous variables presented as mean ± standard deviation (SD) and categorical variables as frequencies and percentages. The association between perinatal risk factors and BERA outcomes was assessed using the Chi-square test or Fisher's exact test for categorical variables. Student's t-test was used to compare the means of continuous variables (*e.g.*, wave V latency) between the normal and abnormal BERA groups. A p-value of <0.05 was considered statistically significant.

## Results:

A total of 150 high-risk infants (82 males, 54.7%; 68 females, 45.3%) were included in the final analysis. The demographic and clinical characteristics of the study cohort are summarized in Table 1 (see PDF). The mean gestational age was 34.2 ± 2.8 weeks and the mean birth weight was 2150 ± 550 grams. Of the 150 infants tested, 45 (30.0%) exhibited abnormal BERA findings, while 105 (70.0%) had normal results. Among the 45 infants with abnormal results, 28 (62.2%) had bilateral impairment and 17 (37.8%) had unilateral impairment. The most common type of abnormality was an elevated hearing threshold suggestive of sensorineural hearing loss (n=36), followed by findings consistent with auditory neuropathy spectrum disorder (absent or abnormal ABR with present OAEs, tested separately) (n=9). The prevalence of various JCIH risk factors among the study population is presented in Table 2 (see PDF). The most frequent risk factors were prematurity (72.0%), NICU stay >5 days (68.7%) and low birth weight (65.3%). The distribution of perinatal risk factors was compared between infants with normal BERA and those with abnormal BERA findings (Table 3 - see PDF). A statistically significant association was observed between abnormal BERA results and the presence of hyperbilirubinemia requiring phototherapy (p<0.001), perinatal asphyxia (p=0.001) and mechanical ventilation for more than 5 days (p<0.001). While a higher proportion of infants with prematurity and low birth weight were in the abnormal BERA group, these associations were not statistically significant (p=0.254 and p=0.312, respectively). Analysis of BERA waveform parameters revealed significant differences between the two groups. Infants in the abnormal BERA group demonstrated a significantly prolonged mean absolute latency for wave V (6.92 ± 0.81 ms) compared to the normal BERA group (5.65 ± 0.34 ms; p < 0.001). The interpeak latency I-V was also significantly longer in the abnormal group (4.88 ± 0.75 ms vs. 4.15 ± 0.29 ms; p < 0.001), suggesting dysfunction in the central auditory brainstem pathways.

## Discussion:

This study aimed to assess the prevalence of auditory dysfunction and its associated risk factors in a high-risk neonatal population using BERA. Our findings reveal a substantial prevalence of abnormal BERA results (30.0%), reinforcing the heightened vulnerability of this cohort to hearing impairments. This prevalence is consistent with figures reported in similar studies from other NICU settings, which typically range from 20% to 40%, but is markedly higher than in the general newborn population [11, 12]. The high prevalence underscores the critical importance of targeted screening programs for NICU graduates, as recommended by the JCIH [3]. The most significant finding of our analysis was the strong and independent association of abnormal BERA outcomes with three key risk factors: severe hyperbilirubinemia, perinatal asphyxia and prolonged mechanical ventilation. The link between hyperbilirubinemia and auditory dysfunction, particularly ANSD, is well-documented [13]. Unconjugated bilirubin is neurotoxic and has a known predilection for the auditory nuclei in the brainstem, potentially disrupting synaptic transmission and leading to abnormal neural synchrony. This is reflected in our BERA findings of prolonged latencies and, in some cases, absent waves, which are classic signs of bilirubin-induced neurologic dysfunction [14]. Our results, showing that 77.8% of infants with abnormal BERA had a history of significant jaundice, strongly support this pathophysiological link and highlight the need for aggressive management of neonatal hyperbilirubinemia. Perinatal asphyxia was another powerful predictor of auditory impairment. The hypoxic-ischemic insult associated with asphyxia can cause widespread neuronal damage throughout the central nervous system. The auditory system, particularly the metabolically active inner hair cells of the cochlea and the complex nuclei of the brainstem, is highly susceptible to oxygen deprivation [15]. The resulting damage can manifest as sensorineural hearing loss or central auditory processing disorders. Our finding that nearly half of the infants in the abnormal BERA group had experienced significant perinatal asphyxia aligns with previous research and emphasizes that a low APGAR score at 5 minutes should be considered a major red flag for potential hearing loss [16]. The third significant factor, mechanical ventilation for more than five days, is often considered a surrogate marker for the overall severity of neonatal illness [17]. Prolonged ventilation is frequently associated with other co-morbidities such as sepsis, hemodynamic instability and prolonged exposure to both ototoxic medications and the high-intensity ambient noise of the NICU environment [18]. While we did not find an independent significant association with ototoxic drug exposure, its effects are likely intertwined with the need for prolonged respiratory support. The combination of these insults likely creates a synergistic effect, increasing the risk of damage to the auditory system [19]. Interestingly, prematurity and low birth weight, while high prevalent in our cohort, were not independently associated with abnormal BERA findings in our statistical analysis. This suggests that it may not be prematurity or low birth weight per se that directly causes hearing loss, but rather the common complications and necessary medical interventions associated with these conditions (such as hyperbilirubinemia, asphyxia and ventilation) that are the primary drivers of auditory pathology [20]. This finding is crucial as it helps to focus clinical attention on the physiological insults suffered by the infant rather than just their gestational age or weight at birth.

## Conclusion:

A high prevalence (30.0%) of auditory dysfunction among high-risk infants admitted to the NICU. Severe hyperbilirubinemia, perinatal asphyxia and the need for prolonged mechanical ventilation were identified as the most significant predictors of abnormal BERA findings. Thus, we show the critical need for robust, targeted hearing screening programs for all NICU graduates, with particular vigilance for infants presenting with these specific risk factors.
